# Long-term outcomes of Guillain-Barré syndrome possibly associated with Zika virus infection

**DOI:** 10.1371/journal.pone.0220049

**Published:** 2019-08-01

**Authors:** Diana M. Walteros, Jesus Soares, Ashley R. Styczynski, Joseph Y. Abrams, Jose I. Galindo-Buitrago, Jorge Acosta-Reyes, Elsa Bravo-Ribero, Zuleima E. Arteta, Alma Solano-Sanchez, Franklyn E. Prieto, Maritza Gonzalez-Duarte, Edgar Navarro-Lechuga, Jorge L. Salinas, Ermias D. Belay, Lawrence B. Schonberger, Inger K. Damon, Martha L. Ospina, James J. Sejvar

**Affiliations:** 1 Instituto Nacional de Salud, Bogota, Colombia; 2 Centers for Disease Control and Prevention, Atlanta, Georgia, United States of America; 3 Stanford University Department of Infectious Diseases, Palo Alto, California, United States of America; 4 TEPHINET - Institute for Global Health, Decatur, Georgia, United States of America; 5 Universidad del Norte, Barranquilla, Colombia; 6 Secretaría de Salud de Barranquilla, Barranquilla, Colombia; 7 University of Iowa Hospitals and Clinics, Iowa City, Iowa, United States of America; Fundacao Oswaldo Cruz, BRAZIL

## Abstract

**Background:**

This prospective cohort investigation analyzed the long-term functional and neurologic outcomes of patients with Zika virus-associated Guillain-Barré syndrome (GBS) in Barranquilla, Colombia.

**Methods:**

Thirty-four Zika virus-associated GBS cases were assessed a median of 17 months following acute GBS illness. We assessed demographics, results of Overall Disability Sum Scores (ODSS), Hughes Disability Score (HDS), Zung Depression Scale (ZDS), and Health Related Quality of Life (HRQL) questionnaires; and compared outcomes indices with a normative sample of neighborhood-selected control subjects in Barranquilla without GBS.

**Results:**

Median age at time of acute neurologic onset was 49 years (range, 10–80); 17 (50%) were male. No deaths occurred. At long-term follow-up, 25 (73%) patients had a HDS 0–1, indicating complete / near complete recovery. Among the group, HDS (mean 1.4, range 0–4), ODSS (mean 1.9, range 0–9) and ZDS score (mean 34.4, range 20–56) indicated mild / moderate ongoing disability. Adjusting for age and sex, Zika virus-associated GBS cases were similar to a population comparison group (n = 368) in Barranquilla without GBS in terms of prevalence of physical or mental health complaints, though GBS patients were more likely to have an ODSS of ≥ 1 (OR 8.8, 95% CI 3.2–24.5) and to suffer from moderate / moderate-severe depression (OR 3.89, 95% CI 1.23–11.17) than the comparison group.

**Conclusions:**

Long-term outcomes of Zika virus-associated GBS are consistent with those associated with other antecedent antigenic stimuli in terms of mortality and ongoing long-term morbidity, as published in the literature. Persons with Zika virus-associated GBS more frequently reported disability and depression after approximately one year compared with those without GBS.

## Introduction

Guillain-Barré syndrome (GBS) is an acute inflammatory polyradiculoneuropathy resulting in limb and cranial nerve weakness, often with respiratory compromise, with variable consequent limitations on physical function. The disease is commonly precipitated by an infection, and infectious agents previously associated with GBS include *Campylobacter jejuni*, *Mycoplasma pneumoniae*, cytomegalovirus, and Epstein-Barr virus.[1] The incidence of GBS worldwide ranges from 0.81 to 1.89 cases per 100,000 person–years,[2] but these estimates may vary depending on robustness of surveillance and the regional prevalence of etiologic triggers. Most recently, the increased incidence of GBS in regions where outbreaks of Zika virus (ZIKV) have occurred epidemiologically suggests that ZIKV is a new infectious agent associated with GBS.[3–7]

GBS is heterogeneous in severity of neurological deficits and prognosis.[8] People with GBS usually experience their most significant weakness within two to four weeks after symptoms begin, and as the disease progresses, weakness can evolve into paralysis. In some cases, the disease can result in neuromuscular respiratory failure leading to the need for ventilatory support. Despite effective therapies, such as intravenous immunoglobulin (IVIG) and plasma exchange, death or disability can occur. Death has been reported to range from 1–18%,[9] and that about 20%-35% of hospitalized patients experience long-term disability.[10, 11]

Studies of GBS outcomes suggest that by one year from onset of the neurologic signs, 62%-92% of patients achieve complete / near complete recovery, regaining the ability to perform manual work and other motor activities.[10–14] However, due to limitations in the physical condition of GBS patients, even years after the acute phase of the disease, their health related quality of life might be compromised by symptoms of fatigue, pain, anxiety, and depression.[15, 16] In addition, most outcomes assessments have been conducted in North America and Europe, where aggressive intervention and rehabilitation services may influence ultimate outcome. Less is known about the eventual outcomes of GBS in resource-limited settings.

Although the prognosis for recovery in GBS patients is generally favorable, with most patients experiencing complete recovery or only minor sequelae, anecdotal reports have suggested that ZIKV-associated GBS has a worse outcome or prognosis than that seen with GBS associated with other antecedent antigenic triggers. However, the long-term neurological and functional outcomes of patients affected by GBS during the ZIKV outbreaks have undergone limited prospective systematic assessments.[6] Therefore, we aimed to analyze the long-term functional and neurologic outcomes of GBS patients associated with ZIKV in Barranquilla, Colombia, an area that experienced a significant outbreak of ZIKV infections and a high incidence of GBS.

## Methods

### Ethics statement

The human subjects review committee at the U.S. Centers for Disease Control and Prevention (CDC) determined this investigation to be public health emergency response and not research, and as such did not require full Institutional Review Board approval. The Colombian National Institute of Health relied on CDC's determination. All subjects provided written informed consent prior to investigation participation.

### Confirmation of GBS status and clinical description

The enrollment process of the patients with GBS for this investigation was previously published by Salinas et al. in 2017.[3] Briefly, in April 2016, the authors searched for case-patients fulfilling Brighton Collaboration criteria for GBS,[17] who were Barranquilla residents according to phone numbers and their residency as listed in their medical record. Forty patients meeting Brighton case definition criteria 1–3 for GBS[18] were included in the investigation, and the demographics and clinical characteristics of these patients were described.[3] All GBS case-patients had laboratory-confirmed or clinically-suspected ZIKV infection; laboratory confirmation by plaque-reduction neutralization testing (PRNT) demonstrated that 5 / 40 had evidence of a previous dengue infection, 21 / 40 had evidence of a previous ZIKV or dengue virus infection, and 1 / 40 had evidence of a recent ZIKV infection. A first assessmenet standardized questionnaire was used to interview the GBS patients to obtain demographic information and antecedent symptoms within a two-month period before the onset of neurological signs/symptoms. Serum samples were collected to perform serologic testing for ZIKV.

**Fig 1:**
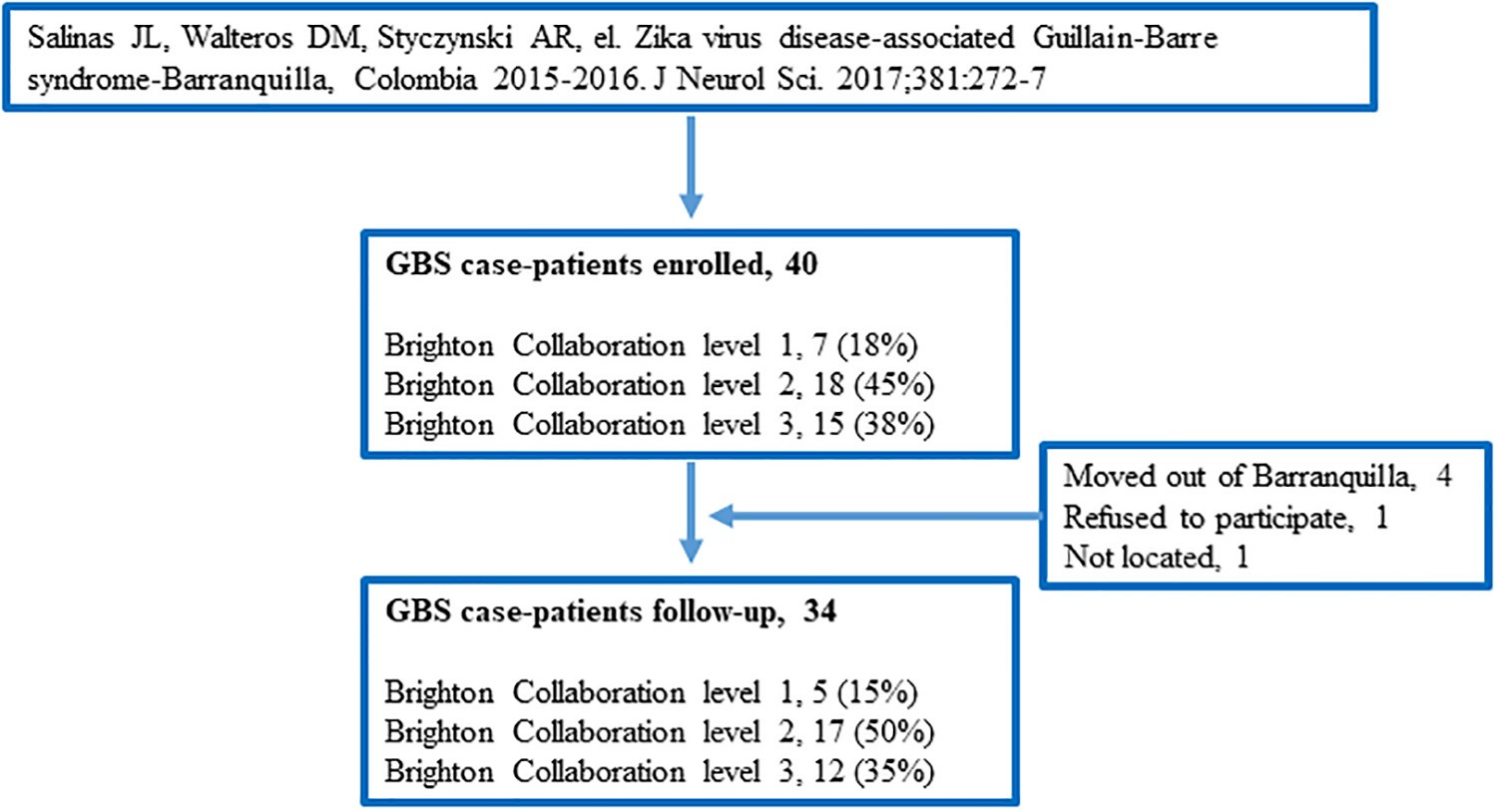
Flow diagram for selection of GBS patients*. *Adapted from Salinas JL, Walteros DM, Styczynski A, et al. Zika virus disease-associated Guillain-Barre syndrome-Barranquilla, Colombia 2015–2016. *J Neurol Sci*. 2017;381:272–277.

In the present study, a follow-up survey to determine long-term disability was conducted between May and July 2017. We re-contacted 34 of the previous 40 patients; four had moved out of Barranquilla, one refused participation, and one was unable to be located. The 34 patients were included in this follow-up investigation (Fig 1).

The patients at follow-up were administered a structured in-person interview to assess functional disabilities, including the Hughes Disability Scale[19] and the Modified Rankin Scale.[20] We also estimated the levels of depression using the Zung Self-Rating Depression Scale;[21] health-related quality of life (HRQoL) was assessed using the CDC Healthy Days questionnaire.[22, 23] A neurologist performed a physical and neurologic exam, which included the determination of each patient’s Overall Disability Sum Score (ODSS).[24]

Because there were no first assessment data for certain indices (ODSS, Zung Depression Scale, HRQoL), we wanted to compare these results from GBS patients to a referent population in order to determine whether there were differences between the GBS group and the general population of Barranquilla. A population sample used for a prior study of health-related quality of life in Barranquilla (Acosta et al., unpublished data) was used for the comparison population. Briefly, 368 non-hospitalized adults ≥ 18 years old who were living at home and were without neurological symptoms were sampled from four geographic regions of the city of Barranquilla in May 2017. The comparison population was selected to be similar in age group and residence to the GBS patient population. The comparison population was assessed for demographic information and measurements of disability, depression and health-related quality of life.

### Statistical analyses

Descriptive statistics were calculated for demographic, clinical, disability, depression, and health-related quality of life data at time of follow up.

Odds ratios were used to compare prevalence of disabilities at the baseline and follow-up surveys. For comparison of cases to the non-hospitalized comparison group, multivariable logistic regression was utilized for questions related to self-reported health status, the health related quality of life, the ODSS, and the Zung scale, adjusting for age and sex. For analyses on individual Zung items, which were answered on a scale of 1 (most normal) to 4 (most depressed), we used proportional odds logistic regression, adjusting for age and sex. For Zung index comparisons, in order to avoid excluding patients who were missing answers for a small number of items, we utilized multiple imputation based on available Zung item answers for each patient using ordered logit modeling from the R package “mice” (multiple imputation by chained equations) [25].

Demographic differences between the case and comparison groups were assessed by the Mann Whitney U test and the chi-squared test with Yates continuity correction.

All data were analyzed using R version 3.3.3 (The R Foundation for Statistical Computing, 2017).

## Results

### Characteristics of GBS cases at baseline and follow-up

The median time from neurologic symptom onset to first assessment survey for the 34 GBS patients was three months (IQR: 2–3 months; range: 0.9–6).[3] In the present study, long-term follow-up data were collected a median of 17 months after symptom onset (IQR: 16–17 months; range: 14–20). The demographics, clinical characteristics, and treatment of these GBS patients at the time of first assessment in 2016 are reproduced in Table 1.

**Table 1:** Descriptive statistics for 34 GBS patients, Barranquilla, Colombia.

Sex		
	Female	17 (50%)
	Male	17 (50%)
Age at onset		
	Median (IQR)	49 (35, 60.75)
	Range	(10, 80)
Days in hospital		
	Median (IQR)	14.5 (9.25, 28.75)
	Range	(4, 60)
Admitted to the ICU/CCU		
	Yes	23 (68%)
	No	11 (32%)
Received mechanical ventilation		
	Yes	7 (21%)
	No	27 (79%)
Received intravenous immunoglobulin		
	Yes	31 (91%)
	No	3 (9%)
Received steroids		
	Yes	13 (38%)
	No	21 (62%)
Received plasma exchange		
	Yes	6 (18%)
	No	28 (82%)
Unable to walk without assistance (at clinical nadir)		
	Yes	13 (41%)
	No	19 (59%)
Unable to walk at all (at clinical nadir)		
	Yes	10 (31%)
	No	22 (69%)

Fifty percent (17/34) of the cohort were male, and the median age at onset of acute GBS was 49 years (IQR 35.0–60.75). The median time between prodromal illness and onset of neurological symptoms was seven days (IQR 2–15). All patients (100%) were admitted to the hospital and median duration of hospitalization was 14.5 days (IQR 9.25–28.75). Sixty-eight percent were admitted to intensive care unit/critical care unit (ICU/CCU) and seven patients (21%) received mechanical ventilation. Treatment received by GBS patients included intravenous immunoglobulin (91%), steroids (38%), and plasma exchange (18%). During acute GBS illness at clinical nadir, 41% of patients were not able to walk without assistance, and 31% were unable to walk at all.

Ninety-one percent of patients went to rehabilitation services after hospital discharge for physical therapy, and 12% received home health therapy (Table 2).

**Table 2:** Characteristics of rehabilitation services for follow-up GBS cases, Barranquilla, Colombia.

Rehabilitation services	N	%
Physical Therapy	31	91
Assistive device	8	24
Home health aid	4	12
Speech Therapy	2	6
**Payment for services**		
Health Insurance	29	85
Personal	2	6
Paid for others	2	6
**Barriers to accessing**		
Referral for service	5	15
No services available	3	9
Transportation/distance	1	3

The neurologic signs demonstrated by the GBS patients at baseline and follow up are summarized in Table 3.

**Table 3:** Characteristics of neurological symptoms at onset and follow-up for confirmed GBS cases, Barranquilla, Colombia.

Disability	Baseline			Follow-up			OR (95%CI) (for disability on follow-up)
	Yes	No	%Yes	Yes	No	%Yes	
Leg weakness	32	2	94%	14	20	41%	0.04 (0.01, 0.21)
Arm weakness	31	3	91%	4	30	12%	0.01 (0.00, 0.06)
Face weakness	18	16	53%	2	32	6%	0.06 (0.01, 0.27)
Leg numbness/ paresthesia	26	8	77%	8	26	24%	0.09 (0.03, 0.29)
Arm numbness/ paresthesia	23	11	68%	4	30	12%	0.06 (0.02, 0.23)

At baseline 94% of these patients reported leg weakness and 91% reported arm weakness. The proportion of patients reporting face weakness, leg numbness, and arm numbness was 53%, 77%, and 68%, respectively. At follow-up the proportion of patients reporting neurological symptoms decreased significantly for all five domains evaluated. Aside from a single patient who reported face weakness at follow-up but not at baseline, all individuals who reported any of these neurologic signs at follow-up had previously reported those signs at baseline.

**Fig 2:**
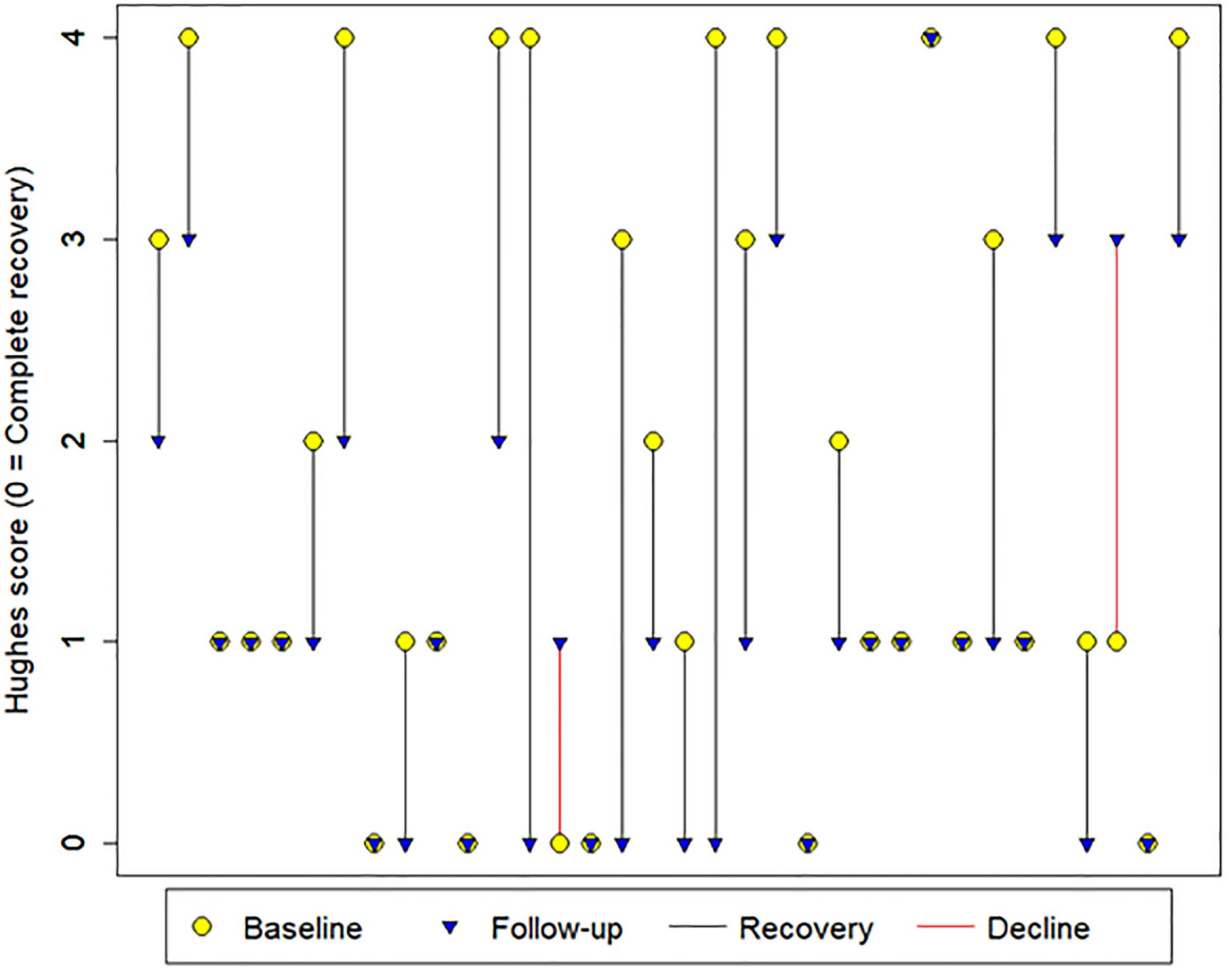
Comparison of Hughes score at baseline and follow-up for 34 GBS patients, Barranquilla, Colombia. The vertical lines connect each patient’s Hughes scores at baseline and follow-up. Eighteen (52%) cases had a Hughes score of 0–1 at baseline and 25 (74%) of cases had a Hughes score of 0–1 at follow-up. Eighteen of the cases had a lower Hughes score at follow-up compared to baseline, 14 had the same Hughes score, and two had a higher Hughes score at follow-up (shown in red). Hughes scores were significantly lower at follow-up (paired T test p-value: 0.0008).

The results of neurologic evaluation using the Hughes score at baseline and follow-up are represented in Fig 2.

**Fig 3:**
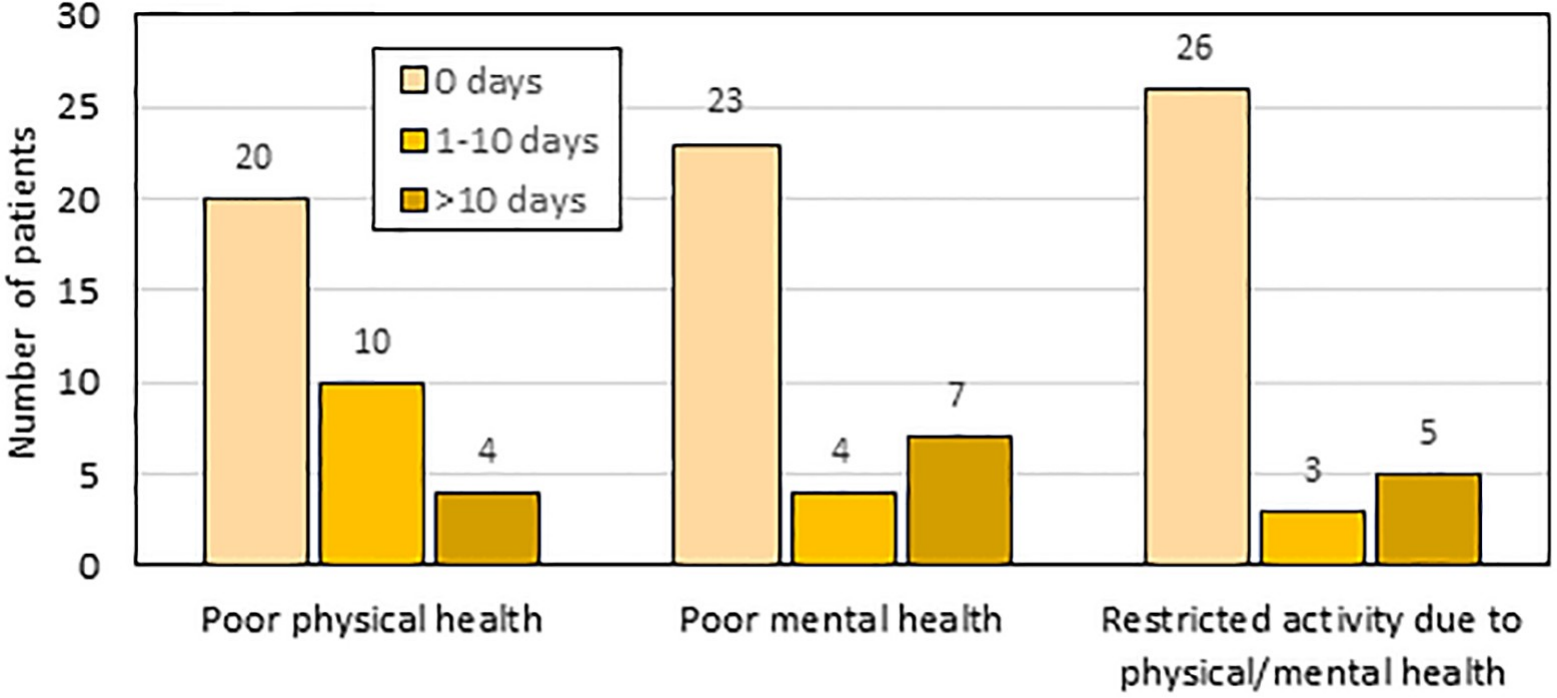
Number of days with physical/mental health issues in previous 30 days. Bar height represents the number of GBS patients with 0 days, 0–10 days, and > 10 days of health issues within the previous 30 days for three different measures of health impairment. Ten patients (29%) reported poor physical health between 1–10 days (in the previous 30 days), while four patients (12%) reported greater than 10 days with poor physical health. Four patients (12%) reported poor mental health between 1–10 days (in the previous 30 days), while seven patients (21%) reported greater than 10 days with poor mental health. Three patients (9%) reported restricted activity due to poor physical or mental health between 1–10 days (in the previous 30 days), while five patients (15%) reported greater than 10 days with restricted activity due to poor physical or mental health.

Ongoing issues with health-related quality of life were captured in the follow-up survey (Fig 3).

**Fig 4:**
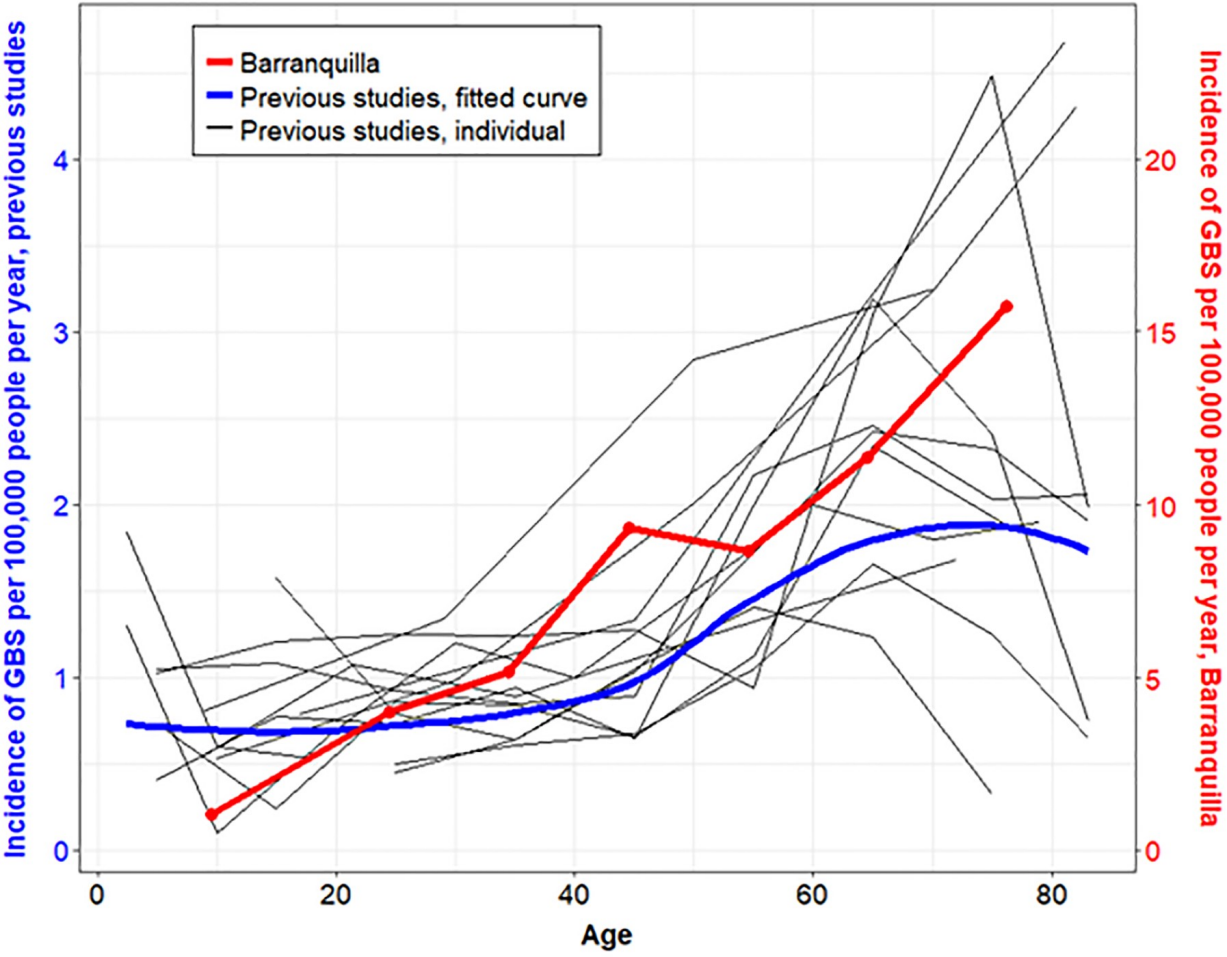
Incidence of GBS by midpoint of age groups, Barranquilla, Colombia, compared to previous studies. The red curve represents age-specific incidence for Barranquilla (right y-axis) and the black curves represent age-specific incidence for 13 previous studies (left y-axis). The blue curve (also left y-axis) represents a loess curve, weighted by person-years, fitted to incidence data from the previous studies. Data for previous studies from Sejvar et al. 2011.[2] While incidence generally increased with age in these previous studies, the observed association between age and incidence was even more pronounced in the Barranquilla investigation.

Incidence of GBS in Barranquilla during the study period was substantially higher than incidence reported in 13 previous studies of GBS incidence conducted in Europe and North America[2] (Fig 4), and comparable to incidence of ZIKV-associated GBS in other areas of the Caribbean and South America. [4, 26]

### Comparison of cases with normal Barranquilla sample population at follow-up

The demographic characteristics of the non-GBS comparison population is shown in Table 4.

**Table 4:** Demographic characteristics of GBS cases and sampled normal population, Barranquilla, Colombia.

Characteristics		Cases	Comparison Group
**Sex**			
	Female	17 (50%)	163 (44%)
	Male	17 (50%)	205 (56%)
**Age (years)**			
	18–24	4 (12%)	53 (14%)
	25–39	7 (21%)	90 (25%)
	40–54	10 (30%)	119 (32%)
	55–69	8 (24%)	58 (16%)
	> 70	5 (15%)	48 (13%)

The non-GBS comparison group had a median age of 44.5 years and an IQR of 30 to 59 years (as compared to a median of 49 years and an IQR of 35 to 60.75 years for cases); the difference in age between the comparison population and cases was not statistically significant (p = 0.33). The proportion of the comparison group that was male (56%) was not significantly different from the proportion of cases that was male (50%) (p = 0.65).

**Fig 5:**
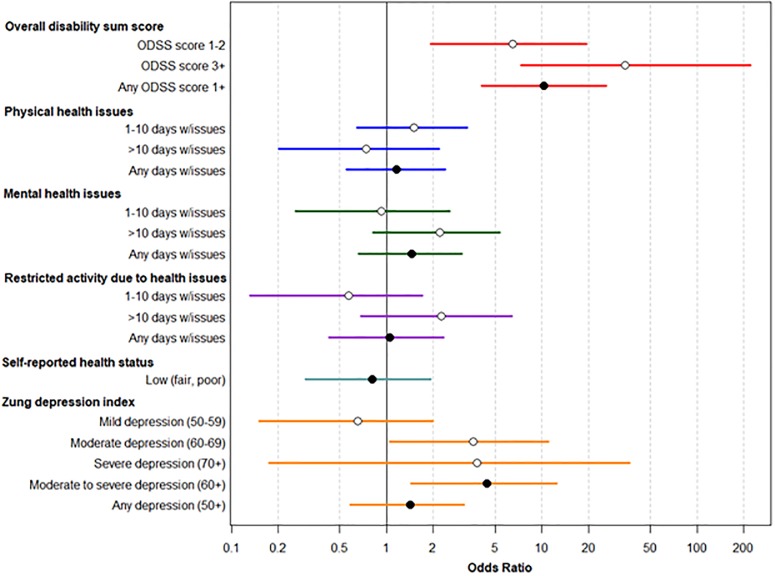
Comparison of disability of GBS cases at follow-up compared to non-GBS comparison group. Odds ratios are adjusted for age and sex. Reference categories for each measure are as following: ODSS (reference score = 0); physical health issues (reference of 0 days in last 30 days with issues); mental health issues (reference of 0 days in last 30 days with issues); restricted activity due to health issues (reference of 0 days in last 30 days with restricted activity); self-reported health status (reference of excellent, very good, or good status); Zung index (reference of score below 50). Empty circles represent comparisons of individual categories with the reference, while black circles represent comparisons of combined categories with the reference. Cases were more likely to have an overall disability sum score (ODSS) of 1 or more (0 indicates no disability) at follow-up compared to the comparison group, adjusted for age and sex. Cases had an odds ratio of 6.48 (95% confidence interval: 1.91, 14.92) of having an ODSS from 1–2 (as opposed to ODSS = 0) compared to the comparison group. The odds ratio for cases having ODSS of 3+ compared to the population comparison group was 34.38 (95% CI: 7.33, 221.95), and the odds ratio for cases having any ODSS score of 1 or more was 10.28 (95% CI: 4.09, 26.09). Cases were more likely to have moderate depression (Zung index between 60–69, as opposed to no depression, Zung index below 50) compared to the population comparison group, with an age- and sex- adjusted odds ratio of 3.81 (95% CI: 1.10, 11.59). Cases were also more likely to have moderate or severe depression (Zung index of 60+) than the population comparison group (odds ratio = 3.89, 95% CI: 1.23, 11.17).

Fig 5 demonstrates comparative data (odds ratios) between cases and comparison group on the various indices assessed.

Table 5 demonstrates these results in tabular form.

**Table 5:** Odds ratios comparing disability for GBS cases at follow-up with the comparison group.

**Physical health issues**	**Case**	**Comparison Group**	**Unadj OR (95% CI)**	**Adj OR (95% CI)**
0 days	20 (59%)	212 (63%)	REF	REF
1–10 days	10 (29%)	72 (21%)	1.47 (0.66, 3.29)	1.50 (0.64, 3.29)
>10 days	4 (12%)	52 (16%)	0.82 (0.27, 2.49)	0.74 (0.20, 2.17)
Any days w/issues	14 (41%)	124 (37%)	1.20 (0.58, 2.45)	1.16 (0.55, 2.37)
**Mental health issues**	**Case**	**Comparison Group**	**Unadj OR (95% CI)**	**Adj OR (95% CI)**
0 days	23 (68%)	252 (76%)	REF	REF
1–10 days	4 (12%)	48 (14%)	0.91 (0.30, 2.76)	0.92 (0.26, 2.54)
>10 days	7 (21%)	33 (10%)	2.32 (0.93, 5.84)	2.20 (0.81, 5.36)
Any days w/issues	11 (32%)	81 (24%)	1.49 (0.70, 3.18)	1.46 (0.66, 3.07)
**Restricted activity due to health issues**	**Case**	**Comparison Group**	**Unadj OR (95% CI)**	**Adj OR (95% CI)**
0 days	26 (77%)	257 (78%)	REF	REF
1–10 days	3 (9%)	51 (16%)	0.58 (0.17, 1.99)	0.57 (0.13, 1.70)
>10 days	5 (15%)	21 (6%)	2.35 (0.82, 6.76)	2.25 (0.68, 6.40)
Any days w/issues	8 (24%)	72 (22%)	1.10 (0.48, 2.53)	1.05 (0.43, 2.34)
**Zung depression index**	**Case**	**Comparison Group**	**Unadj OR (95% CI)**	**Adj OR (95% CI)**
Normal (<50)	24 (71%)	293 (80%)	REF	REF
Mild depression (50–59)	4 (12%)	58 (16%)	0.84 (0.28, 2.52)	0.79 (0.22, 2.17)
Moderate depression (60–69)	5 (15%)	14 (4%)	4.36 (1.45, 13.13)	3.81 (1.10, 11.59)
Severe depression (70+)	1 (3%)	3 (1%)	4.07 (0.41, 40.63)	4.52 (0.20, 46.78)
Moderate to severe depression (60+)	6 (18%)	17 (5%)	4.31 (1.55, 11.94)	3.89 (1.23, 11.17)
Any depression (50+)	10 (29%)	75 (20%)	1.63 (0.75, 3.55)	1.48 (0.63, 3.24)
**Zung index, emotional (12 questions)**	**Case**	**Comparison Group**	**Unadj OR (95% CI)**	**Adj OR (95% CI)**
Normal (<30)	24 (71%)	315 (86%)	REF	REF
High (30+)	10 (29%)	53 (14%)	2.48 (1.12, 5.47)	2.31 (0.97, 5.22)
**Zung index, physical (8 questions)**	**Case**	**Comparison Group**	**Unadj OR (95% CI)**	**Adj OR (95% CI)**
Normal (<20)	24 (71%)	279 (76%)	REF	REF
High (20+)	10 (29%)	89 (24%)	1.31 (0.60, 2.84)	1.18 (0.51, 2.56)
**ODSS**	**Case**	**Comparison Group**	**Unadj OR (95% CI)**	**Adj OR (95% CI)**
Normal ODSS (0)	22 (65%)	346 (94%)	REF	REF
ODSS score 1–2	5 (15%)	13 (4%)	6.05 (1.98, 18.50)	6.48 (1.91, 19.42)
ODSS score 3+	7 (21%)	9 (2%)	12.23 (4.16, 35.94)	34.38 (7.33, 221.95)
Any score 1+	12 (35%)	22 (6%)	8.58 (3.76, 19.57)	10.28 (4.09, 26.09)

All odds ratio are the relative odds of a given category as opposed to the reference (healthy) category for the cases compared to those odds for the comparison group. Adjusted odds ratios are from logistic regression controlling for sex and age. Zung scores: only 34 of 8040 (0.4%) of responses were missing, but 27 of 402 (6.7%) of individuals were missing at least one response, so multiple imputation was used to account for missing values.

## Discussion

Our investigation evaluated the long-term outcomes of ZIKV-associated GBS at 14–20 months after initial GBS illness. Our findings suggest that the long-term morbidity in ZIKV-associated GBS are consistent with that described in GBS literature for GBS associated with other antecedent antigenic stimuli. The proportion of patients requiring mechanical ventilation during hospitalization (20%) is consistent with estimations in other GBS assessments.[27–30] The proportion of patients experiencing complete or near-complete recovery at time of hospital discharge (Hughes disability score 0–1, 73%) is also consistent with previously published values; review articles summarizing the long-term outcomes of GBS due to other antecedent triggers have consistently provided such estimates.[31–34] In terms of long-term outcomes, nearly all patients continued to experience improvement in limb and cranial nerve weakness during the intervening 14–20 months, as evidenced by improving Hughes scores and proportional improvement in limb weakness, suggesting that improvement from ZIKV-associated GBS may continue for months following hospital discharge. Other long-term assessments of recovery following GBS have found similar evidence of ongoing improvement in strength and function for months or years following acute GBS illness.[10–12, 35, 36]

At long-term assessment, we found that GBS patients were more likely than the comparison population group to have ongoing disability as evidenced by an ODSS of 1 or greater as assessed by neurologic examination; this is not necessarily a surprising finding, but does demonstrate ongoing morbidity in the GBS group. GBS patients were also more likely to experience moderate or moderate-severe depression than the general population of Barranquilla, conceivably due to a reactive depression after experiencing a disabling illness. However, on quality of life indices assessing the occurrence of physical or mental difficulties or functional problems due to physical or mental difficulties in the 30 days prior to interview, there were no substantial differences between the GBS patients and the comparison population group. This suggests that, despite ongoing weakness and depression in some, overall quality of life indices did not differ significantly between those with and without GBS in this population.

The proportion of patients with complete or near-complete recovery at follow-up (Hughes score 0–1, n = 25, 73%) is similar to other investigations assessing the outcomes of Zika-associated GBS.[6, 37] However, recovery was not universal, and nine patients continued to experience function-limiting sequelae at follow-up. These sequelae were predominantly in the domains of ongoing weakness and depression.

Other investigations assessing long-term (one year or greater) outcomes of GBS have had variable findings. Cheng et al. determined that 40% of 60 GBS patients remained with physical and functional sequelae at one year.[38] Alternately, another retrospective investigation into the long-term outcomes of ZIKV-associated GBS determined that 80% of 15 GBS patients experienced good recovery at one year, with 13% having physical / functional sequelae.[37] Our investigation demonstrated recovery patterns consistent with that of other published data assessing GBS attributable to etiologies other than ZIKV.[39, 40]

As previously demonstrated, incidence of GBS increased with age.[2] However, the overall incidence in nearly every age group was far in excess of what would normally be expected, particularly in persons over age 60. A similar phenomenon was witnessed in Brazil, where age-specific incidence from a ZIKV-associated GBS case-control investigation demonstrated substantially higher incidence rates among all age groups and particularly in persons aged 60 or older.[4] The reason for the dramatic increased incidence among older age groups in ZIKV-associated GBS is unclear.

Our investigation has limitations. Not all cases were laboratory-confirmed ZIKV infections; a proportion of cases were diagnosed using clinical criteria; thus, misclassification of some cases may be possible. However, all cases met clinical compatibility criteria for ZIKV infection as published elsewhere.[41] The fact that the first assessment was conducted up to 6 months following onset of neurologic illness may have resulted in recall bias in some individuals. We were unable to assess risk factors or other prognostic factors associated with clinical outcomes, which would have been a valuable asset to this investigation. However, we were able to provide robust, prospective, systematic assessments on a relatively large group of ZIKV-associated GBS cases to determine long-term outcomes, an important strength of our investigation. In addition, this study was able to compare physical, mental, and quality of life indices with a normative population. Compared to other investigations of outcomes of ZIKV-associated GBS,[6, 37] ours was a prospective investigation covering over a year of follow-up.

## Conclusions

In summary, long-term outcomes of ZIKV-associated GBS are consistent with GBS presentations associated with other etiologies in terms of mortality and ongoing long-term morbidity, as published in the literature. Additionally, long-term physical and mental status among persons with ZIKV-associated GBS after one year were similar to persons without GBS, though disability and depression were more common among GBS patients after one year. Further long-term clinical and epidemiologic assessments of ZIKV-associated GBS using larger sample sizes are needed to substantiate these findings and identify prognostic indicators for outcome.
